# CB2R Deficiency Exacerbates Imiquimod-Induced Psoriasiform Dermatitis and Itch Through the Neuro-Immune Pathway

**DOI:** 10.3389/fphar.2022.790712

**Published:** 2022-01-31

**Authors:** Li Li, Xin Liu, Wenqiang Ge, Chao Chen, Yuqiong Huang, Zilin Jin, Muouyang Zhan, Xiaoru Duan, Xinxin Liu, Yi Kong, Jian Jiang, Xuemei Li, Xin Zeng, Fei Li, Shibin Xu, Man Li, Hongxiang Chen

**Affiliations:** ^1^ Department of Dermatology, Union Hospital, Tongji Medical College, Huazhong University of Science and Technology, Wuhan, China; ^2^ Department of Neurobiology, School of Basic Medicine, Tongji Medical College, Huazhong University of Science and Technology, Wuhan, China; ^3^ Department of Rheumatology, Union Hospital, Tongji Medical College, Huazhong University of Science and Technology, Wuhan, China; ^4^ Department of Dermatology, Union Shenzhen Hospital, Huazhong University of Science and Technology, Shenzhen, China; ^5^ Department of Dermatology, The 6th Affiliated Hospital of Shenzhen University Health Science Center, Shenzhen, China

**Keywords:** psoriasis, the cannabinoid receptor 2, inflammation, itch (pruritus), nerve fiber

## Abstract

**Background:** Cannabinoid receptor 2 (CB2R) is a potential target for anti-inflammatory and pain therapeutics given its significant immunomodulatory and analgesic effects. However, the role of CB2R in imiquimod (IMQ)-induced psoriasiform dermatitis (PsD) and itch is poorly understood.

**Objective:** To investigate the function and mechanism of CB2R in PsD and itch in mice.

**Methods:** Following daily treatment with topical IMQ cream for 5-7 consecutive days in C56BL/6 wild-type (WT) and CB2R gene knockout (KO) mice, we assessed the Psoriasis Area and Severity Index (PASI) scores and the scratch bouts every day, and hematoxylin and eosin (H&E) staining, toluidine blue staining were used to observe the histological changes. mRNA levels were analyzed by quantitative real-time polymerase chain reaction (qRT-PCR). Protein levels were detected by western blotting (WB), immunohistochemistry (IHC), immunofluorescence (IF) and cytometric bead array (CBA). Flow cytometry (FCM) was used to examine the proportion of Th17/Treg cells.

**Results:** We found that CB2R expression levels were increased in mice with psoriasis. Compared with WT mice, CB2R deficiency exacerbated IMQ-induced PsD and scratching bouts and upregulated the expression of proinflammatory cytokines by increasing the infiltration of CD4^+^ T cells and the Th17/Treg ratio. Obvious proliferation and prolongation of nerve fibers and high expression of nerve growth factor (NGF) were observed in PsD and CB2R KO mice. Pretreatment with the CB2R agonist, JWH-133 significantly reversed inflammation and scratching bouts. CB2R didn't participate in the induction of itch in psoriasis by regulating the expression of IL-31, thymic stromal lymphopoietin (TSLP) and mast cells in mouse skins.

**Conclusion:** Our results demonstrate that CB2R plays a pivotal role in the pathophysiology of psoriasis, providing a new potential target for anti-inflammatory and antipruritic drugs.

## Introduction

Psoriasis is a chronic, immune-mediated skin disease caused by the interaction of multiple factors. The most characteristic skin lesions of psoriasis are erythematosus plaques covered with silvery scales, and a greatly thickened epidermis with elongated rete ridges into the dermis (Julià et al., 2012; Boehncke and Schön, 2015). In addition, it has been reported that approximately 70–90% of patients with psoriasis suffer from itch (Szepietowski and Reich, 2016), which can easily be ignored by clinicians. It is currently widely accepted that the pathophysiology of psoriasis is related to the activation and dysregulation of the innate and adaptive immune systems. Various immune cells secrete cytokines, which can directly or indirectly aggravate or even induce itch by increasing the inflammatory response (Komiya et al., 2020). Given the lack of available treatments for psoriatic patients, it is urgent to develop more effective and safer anti-inflammatory and antipruritic therapies.

Cannabinoids are chemical substances that exert their therapeutic effects by binding to cannabinoid receptors. Cannabinoid receptor-2 (CB2R) is a G-protein-coupled receptor and is primarily located on immune organs and cells (Munro et al., 1993; Galiègue et al., 1995). Studies have shown that CB2R is involved in the pathophysiological processes of skin cell proliferation and differentiation, immune regulation, and sensory conduction (Sheriff et al., 2020). In addition, CB2R activation exerts analgesic effects and inhibits the scratching behaviour of rodents (Phan et al., 2010; Haruna et al., 2015). Thus, CB2R agonists may act as potential candidates for new anti-inflammatory and antipruritic drugs by interfering with the occurrence and progression of inflammatory skin diseases. However, to date, it remains unknown whether targeting CB2R can alleviate inflammation and itch in psoriasis. The molecular biological mechanism of its antipruritic effect also needs further study.

Regulatory T cells (Tregs) regulate the inflammatory response in autoimmune diseases and are important in maintaining immune tolerance (Kanda et al., 2021). Peripheral Tregs and Th17 cells are formed by the differentiation of CD4^+^ T cells, of which Tregs are differentiated depending on the activity of forkhead/winged helix transcription factor (FOXP3), which is regulated by transforming growth factor-β (TGF-β) or IL-2 (von Knethen et al., 2020). Th17 cells are differentiated according to retinoid-related orphan nuclear receptor γt (RORγt) activity regulated by TGF-β, IL-1 or IL-6 (Pandiyan and McCormick, 2021). The functional defect of Tregs in psoriasis cannot sufficiently inhibit the proliferation of Th17 cells or inflammatory cytokines production, which may lead to the development and exacerbation of psoriasis. Compared with healthy skin, the ratio of Th17/Treg cells is higher in psoriatic skin lesions. Therefore, we selected Th17/Treg cells as the main research object.

Chronic itch is associated with increased levels of nerve growth factor (NGF). NGF belongs to the neurotrophic factor family (Yamaguchi et al., 2009) and influences the inflammatory reaction. On the other hand, NGF causes the elongation and branching of epidermal nerve fibers, which leads to hypersensitivity of itch and inflammation aggravation in psoriasis (Jaworecka et al., 2021). However, previous studies yielded contradictory results. Some researchers have observed that compared with nonpruritic skin, NGF content was increased in pruritic psoriasis skin lesions (Yamaguchi et al., 2009) accompanied by increased nerve density in the skin (Tan et al., 2019), whereas others did not see such a correlation (Taneda et al., 2011). Further studies are needed to clarify its exact role in psoriasis.

To that end, this study sought to investigate whether CB2R deficiency modifies skin nerve fibers hyperreactivity and the itch-related scratching observed in psoriatic mice by regulating NGF. This study also assessed the role and mechanism of CB2R in IMQ-induced psoriasis inflammation and itch.

## Materials and Methods

### Chemicals and Reagents

AM-630 (A3168), JWH-133 (B7941) were purchased from APExBIO (United States). Dimethyl- sulfoxide (DMSO) was purchased from Sigma. AM-630 and JWH-133 were dissolved in 10% DMSO, 20% Tween-80 and 70% saline. Mice were injected intraperitoneally with AM-630 (1/3/5 mg/kg/day), JWH-133 (1/3/5 mg/kg/day), and intradermally with them 2.5 mg/kg/day respectively, or an equal volume of solvent (control group) every day.

### Human Sample Collection

Skin sample were obtained through surgical biopsy from Union Hospital, Tongji Medical College of Huazhong University of Science and Technology in Wuhan, China. All patients involved signed informed consent. The research protocols conformed to the declaration of Helsinki Principles and were approved by the medical ethics committee of Tongji Medical College of Huazhong University of Science and Technology.

### Animals

All the animal experimental protocols in this study were conducted in accordance with the ARRIVE guidelines and were approved by the Institutional Animal Care and Use Committee of Tongji Medical College of Huazhong University of Science and Technology (Wuhan, China). Male CB2R^−/-^ mice on C57BL/6 background and C57BL/6 wild-type mice aged 6–8 weeks were purchased from Jackson Laboratories. Experimental groups consisted of five mice each and the studies were repeated three times.

### Induction of Psoriasiform Skin Inflammation by Imiquimod

A daily topical dose of 62.5 mg of commercially available imiquimod cream (5%) (Mingxin Pharmaceuticals, Sichuan, China) was applied to the shaved back skin (2.5 cm × 2 cm) of mice for seven consecutive days (days 0–6) (Moos et al., 2019). Control mice were treated similarly with vaseline jelly as the control vehicle cream. Disease severity was assessed by using a scoring system based on the clinical Psoriasis Area and Severity Index (PASI). To be precise, erythema, scaling, and thickening were scored independently on a scale from 0 to 4 (0, none; 1, slight; 2, moderate; 3, marker; 4, very marked), and the cumulative score was used as a total score (scale 0–12). Disease severity was assessed by two researchers in a blinded manner. On day 8, serum samples and skin tissues were taken from the sacrificed mice for subsequent studies.

### Behavioral Scratching Testing

The behavioral scratching was recorded by video. Mice were habituated individually to an observation chamber for 30 min before testing for five consecutive days. Twenty to 22 hours after each topical application of IMQ, mice were videotaped from below for 60 min. The number of videotaped scratch bouts was counted by two trained observers blinded to the treatment condition. A scratch bout was defined as one or more rapid back-and-forth hind paw motions directed toward and contacting the treated area or scratched directly at the area around the injection site continuously for any length of time and lasted, ending with licking or biting of the toes or placement of the hind paw on the floor (Sakai et al., 2016). The use of each fore paw was classified as grooming behavior and was not considered scratching.

### Histology and Immunohistochemistry

Animals were euthanized under sodium pentobarbital anesthesia, and the skin was acutely dissected on the ice. Skin was fixed in 4% paraformaldehyde, embedded in paraffin and cut in 15-μm sections on a microtome. The sections were partly stained with hematoxylin and eosin (H&E) or toluidine blue, or prepared for rabbit monoclonal anti-CD4 (1:1000, Abcam) for IHC. Means of epidermal thickness, the number of mast cells and the degranulation mast cell from toluidine blue staining were calculated based on five randomly selected fields of view per mouse as previously described (Liu et al., 2017). And stained cells were counted under high original magnification (×200) power fields of a light microscope by ImageJ software. Each section was examined independently by two investigators in a blinded manner.

### Immunofluorescence Analysis

Skin was fixed in 4% paraformaldehyde for 24 h and dehydrated with 30% sucrose, frozen in optimal cutting temperature (OCT) compound (Tissue-Tek, Sakura Finetek, Torrance, CA), and cut in 15-μm sections on a cryostat. The sections were incubated with 5% donkey serum and then immunofluorescent staining with a rabbit CB2R (1:200, Abcam, Cambrige, United Kingdom), PGP 9.5 (1:500, ABclonal), c-kit (1:500, RD system) at 4°C overnight, followed by incubation with the corresponding secondary antibody conjugated with Alexa Fluor 594 (1:500; Jackson Laboratory) at 37°C for an hour. All sections were counterstained with 4′,6-diamino-2-phenylindole (DAPI) at room temperature for 5 min. Images were captured from 3-4 skin sections from each animal and were imaged at 20× magnification.

The PGP 9.5-immunoreactive nerve fibers crossing the epidermis from the dermis were quantified by IENFD (expressed as fibers/linear mm) as described previously (Gylfadottir et al., 2022).

### Western Blotting

Total proteins samples from the mouse tissues were extracted using lysis buffer for 30 min and the lysates were centrifuged at 12,000 rpm for 15 min at 4°C. The concentrations of proteins in the supernatants were measured using a BCA protein assay kit. Equal amounts of protein were separated by SDS-PAGE gel electrophoresis and then transferred to PVDF membranes. After blocking with 5% skim milk powder for 1 h at room temperature, the membranes were incubated with primary antibodies against CB2R (1:500, ABclonal), NGF (1:500, Affinity) and β-actin (1:20,000, ABclonal) at 4°C overnight. Secondary antibodies were labeled with horseradish peroxidase and incubated for 1 h at room temperature. The antigen-antibody complexes formed were detected by enhanced chemiluminescence in accordance with the manufacturer’s instructions. Band signal intensities of the western blot films were assessed using the ImageJ software. Relative protein expression levels were normalized to that of β-actin (internal control).

### RNA Isolation and Quantitative Real-Time PCR

RNA isolation was performed using Trizol reagent (Vazyme Biotech Co. Ltd, Nanjing, China). Conversion of total RNA to cDNA was performed with cDNA Reverse Transcription Kit (Vazyme Biotech Co. Ltd, Nanjing, China). All real-time PCR reactions were performed using the Real Time PCR System and the quantitative reverse-transcription PCR assay was carried out using SYBR Green PCR Master Mix (Vazyme Biotech Co. Ltd, Nanjing, China) on StepOne v2.3 detection system (Life Technologies, CA, United States). Forty cycles of amplification were performed involving sequential denaturation at 95°C for 30 s, annealing at 60°C for 5 s, and extension at 72°C for 30 s. The messenger RNA (mRNA) levels were normalized to those of the β-actin gene. Real-time PCR analysis was performed using the 2^-△△CT^ method. All primer pairs are listed in Table 1.

**TABLE 1:** Primer sequences for real-time quantitative PCR.

Name	Forward 5′ - 3′	Reverse 5' - 3′
CB2	CGT​GAT​CTT​CGC​CTG​CAA​CT	GTC​AAC​AGC​GGT​TAG​CAG​CA
β-actin	TGG​CAC​CCA​GCA​CAA​TGA​A	TAA​GTC​ATA​GTC​CGC​CTA​GAA​GCA
GAPDH	TCTCCTGCGACTTCAACA	TGT​AGC​CGT​ATT​CAT​TGT​CA
NGF	AAGGCTTTGCCAAGGACG	GTGATGTTGCGGGTCTGC
IL-1β	CTTCAGGCAGGCAGTATC	CAG​CAG​GTT​ATC​ATC​ATC​ATC
TNF-α	TGT​CCA​TTC​CTG​AGT​TCT​G	GGAGGCAACAAGGTAGAG
IL-17A	ACTACCTCAACCGTTCCA	GAA​TCT​GCC​TCT​GAA​TCC​A
IL-17F	TGC​TAC​TGT​TGA​TGT​TGG​GAC	AAT​GCC​CTG​GTT​TTG​GTT​GAA
IL-17C	ATGCTTGTGTCGTGGATG	GTGCCTGGAATGTCTGTC
IL-23A	ACCTGCTTGACTCTGACA	CCA​CTG​CTG​ACT​AGA​ACT​C
IL-10	TTT​GAA​TTC​CCT​GGG​TGA​GAA	ACA​GGG​GAG​AAA​TCG​ATG​ACA
IL-6	CCG​CTA​TGA​AGT​TCC​TCT​C	GGT​ATC​CTC​TGT​GAA​GTC​TC
CXCL-1	CTG​GGA​TTC​ACC​TCA​AGA​ACA​TC	CAG​GGT​CAA​GGC​AAG​CCT​C
CCL-20	GCC​TCT​CGT​ACA​TAC​AGA​CGC	CCA​GTT​CTG​CTT​TGG​ATC​AGC
TGF-β	TTG​CTT​CAG​CTC​CAC​AGA​GA	TGG​TTG​TAG​AGG​GCA​AGG​AC
ROR-γt	GTG​GAG​CAG​AGC​TTA​AAC​CCC	ATG​ACT​GAG​AAC​TTG​GCT​CCC
Foxp-3	CAC​CTA​TGC​CAC​CCT​TAT​CCG	CAT​GCG​AGT​AAA​CCA​ATG​GTA​GA
IL-31	TCA​GCA​GAC​GAA​TCA​ATA​CAG​C	TCG​CTC​AAC​ACT​TTG​ACT​TTC​T
TSLP	ACT​GCC​ATG​ATG​AGG​TGG​TC	GTC​CTG​GGT​CTG​AAC​CCT​TT

### Flow Cytometry Analysis

Spleens from the individual mice were freshly isolated and mechanically dissociated. RBCs were lysed with lysis buffer (Biosharp, Beijing Labgic Technology Co. Ltd). Single cell suspensions of spleen were passed through a 40-μm sterile wire screen. Cell suspensions were washed twice in RPMI 1640 (Gibco, ThermoFisher Biochemical Products Co. Ltd) and stored in media containing 10% FBS on ice until used within 2 h. Splenocytes were stimulated with 50 μL phorbol 12-myeistate 13-acetate (PMA) (1 μg/ml), 40 μL ionomycin (50 μg/ml), and 20 μL monensin (0.1 mg/ml) for 5 h at 37°C humidified incubator. For cell surface antigen staining, cells were pre-blocked for Fc receptors (BD, Pharmingen) for 15 min at 4°C. The cells were then washed with staining buffer following by staining with Abs for 1 h at 37°C. The cells were washed with staining buffer, then resuspended in 300 μL of staining buffer. Flow cytometry was carried out using a FACS Canto Ⅱ flow cytometer (BD) and the ratio of Th17 cells and Treg cells were analyzed. All the antibodies information were listed in Supplementary Table S1.

### Cytometric Beads Array for Detecting Mouse Th1/Th2/Th17 Cytokines

The content of Th1/Th2/Th17 cytokines including IL-2, IL-4, IL-6, IL-10, TNF-α, IFN-γ and IL-17A concentrations was measured by the CBA kits (BD, Pharmingen, United States) according to the manufacturer’s protocols. Data were acquired on FACS Canto-II flow cytometer (BD Bioscience, United States) and analyzed using FCAP Array software (BD Bioscience).

### Statistics

The data were expressed as mean ± SD and analyzed by Graph Prism 8.2 software (GraphPad Software, San Diego, CA, United States). One-way analysis of variance (ANOVA) analyses was used to evaluate changes in groups by SPSS 25.0 software (SPSS Software, United States). Results were considered statistically significant if *p values were <0.05, **p values were <0.01 or ***p values were <0.001 between the control and experimental groups.

## Results

### CB2R Deficiency Exacerbated Psoriasiform Dermatitis Induced by IMQ Treatment

**FIGURE 1:**
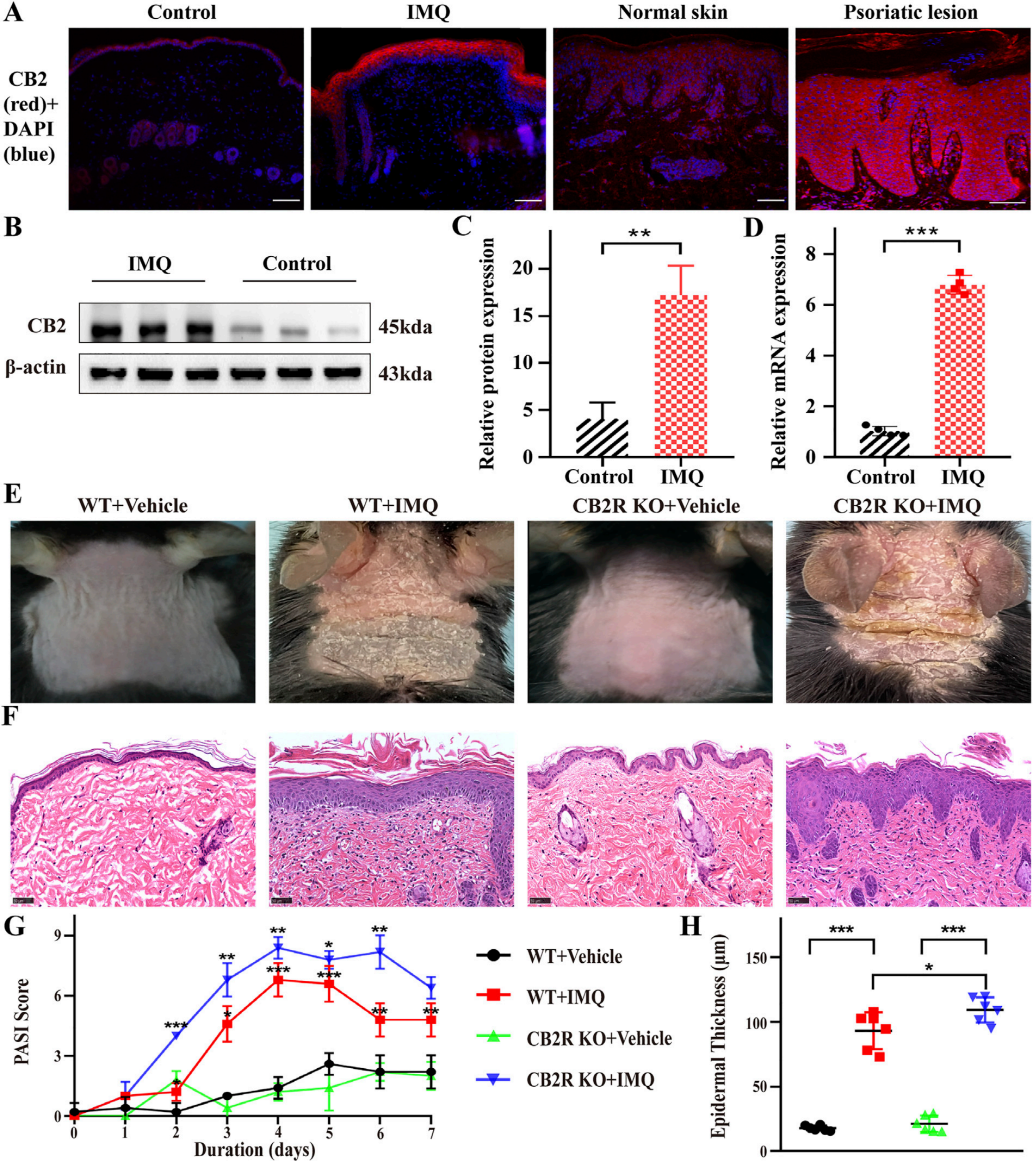
CB2R was upregulated throughout the epidermis, and CB2R deficiency exacerbated the morphological and histological features of psoriasiform dermatitis induced by IMQ treatment. **(A)** Immunofluorescence was used to detect the protein level of CB2R in psoriasiform dermatitis, human normal skin and psoriasis lesions (scale bar: 100 μm). blue, DAPI; red, CB2R. **(B, C)** Protein levels of CB2R were quantified by western blotting. β-actin was used as a loading control. Relative protein expression was normalized to that of the internal control. **(D)** mRNA levels of CB2R in skin tissues from the normal and psoriasis groups were analyzed by real-time quantitative PCR. **(E)** Representative phenotype manifestation of WT and CB2R KO mouse back skin induced by IMQ application at day 7. **(F)** H&E staining of cross-sectional slices of the dorsal skin of C57BL/6 mice on the seventh day. Scale bar represents 50 μm. **(G)** Clinical scores for disease severity were calculated daily of epidermal erythema, scales, and thickening of the dorsal skin. The PASI score was calculated by adding the scores of three independent criteria (ranging from 0 to 12). **(H)** The epidermal thickness of the dorsal skin on the seventh day was measured by four randomly selected fields per section of each mouse. Results are representative of three independent experiments. Error bars represent mean ± SD. (*n* = 5 for each group). *p < 0.05, **p < 0.01, and ***p < 0.001 when compared.

Although major CB2R immunoreactivity in basal keratinocytes of the epidermis in human skin has been observed (Ständer et al., 2005), reports about CB2R expression in PsD and human psoriasis lesions are lacking. We first analyzed CB2R localization in psoriatic skin tissues by immunofluorescence staining. We observed that the fluorescence intensity of CB2R in the epidermis of psoriatic lesions was greater than that of normal skin. These cells were mainly located in the cytoplasm of the epithelial cells and diffusely distributed in the suprabasal keratinocytes of the epidermal stratum spinosum and stratum granulosum in psoriasiform skin and psoriatic lesion biopsies (Figure 1A). Strong positive staining for CB2R was also observed in the dermis in human compared with negative control (Supplementary Figure S1). Next, we measured CB2R protein and mRNA expression levels in psoriasiform skin inflammation and normal skin tissues using WB and qRT-PCR analysis. Figures 1B–D showed that CB2R levels were significantly increased in the IMQ group compared with the normal control group. These data showed dysregulation of CB2R in psoriasis.

Then, we investigated the clinical and histopathological features of WT and CB2R KO mice following IMQ treatment. CB2R KO mice exhibited worse clinical outcomes with more severe erythema and scales and increased skin thickness by IMQ application than WT mice (Figure 1E). Hematoxylin-eosin (H&E) staining performed on the dorsal skin of the mice on Day 7 revealed more severe epidermal hyperplasia, significantly increased acanthosis and parakeratosis and more intense inflammatory cell infiltration in CB2R KO mice than in WT mice after IMQ application (Figure 1F). Consistently, the average Psoriasis Area and Severity Index (PASI) scores of the four groups were graded daily to assess their clinical changes. Compared with WT mice, the total PASI scores of CB2R KO mice were increased after IMQ application (Figure 1G). We measured epidermal thickness with ImageJ analysis of H&E skin sections. Epidermal hyperplasia was significantly increased in CB2R KO mice compared with WT mice (Figure 1H). Thus, CB2R deficiency exacerbated IMQ-induced PsD.

### CB2R Deficiency Increased CD4^+^ T Cells Infiltration and Proinflammatory Cytokines Expression in IMQ Mice

**FIGURE 2:**
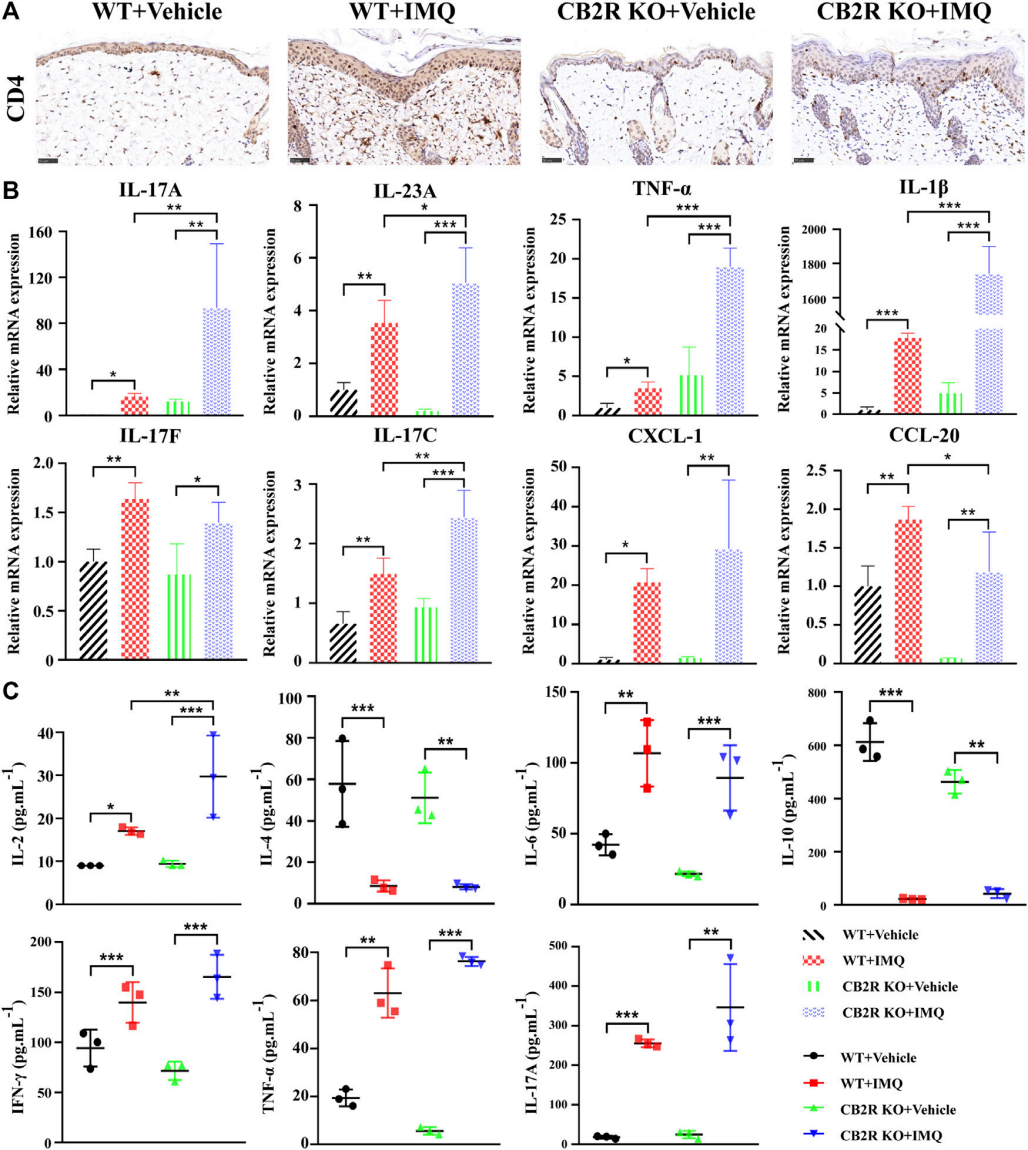
Effects of CB2R on the infiltration of CD4^+^ T cell and expression levels of inflammatory cytokines in mice with IMQ-induced psoriasiform dermatitis. **(A)** The representative immunohistochemical staining images of IMQ- and vehicle- treated skin in WT and CB2R KO mice at day 7. Scale bar represents 50 μm.**(B)** Real-time quantitative PCR was performed to quantify the mRNA level of IL-17A, IL-23A, TNF-ɑ, IL-1β, IL-17F, IL-17C, CXCL-1, CCL-20 in the skin biopsies from mice. Data are obtained from duplicate samples from five mice in each group. **(C)** CBA assay was performed to quantify the protein level of IL-2, IL-4, IL-6, IL-10, IFN-γ, TNF-ɑ and IL-17A in the serum from mice. All the assays were repeated three times with consistent results. Error bars represent mean ± SD. *p < 0.05, **p < 0.01, and ***p < 0.001 when compared.

In both psoriasis and relevant mouse models, the IL-23/Th17 axis and Th1/Th2/Th17 balance play a major role in disease (Gauld et al., 2019). To appraise the effects of CB2R on IMQ-induced local and systemic inflammation, we used IHC to observe the infiltration of CD4^+^ T cells, which are the main source that induce differentiation into various subtypes of T cells in psoriasis. The numbers of CD4^+^ T cells in IMQ-treated CB2R KO mice were significantly increased compared with those in IMQ-treated WT mice (Figure 2A and Supplementary Figure S2A). We also examined the mRNA expression of key inflammation genes that are known to regulate psoriatic skin inflammation. As shown in Figure 2B, relatively increased mRNA levels of IL-17A, IL-23A, TNF-α, IL-1β, and IL-17C were noted in the psoriasiform skin lesions of CB2R KO mice as compared with WT mice. Although the expression of IL-17F, CXCL-1, and CCL-20 in IMQ-treated CB2R KO PsD mice was higher than that in CB2R KO vehicle mice, there was no significant difference in PsD between CB2R KO mice and WT mice, and CCL-20 mRNA levels were somewhat lower in the two groups. In addition, the CBA assay showed that the levels of Th1 (IL-2, IFN-γ, TNF-α) and Th17 (IL-17A) cell-related cytokines in the serum were markedly increased, whereas the levels of Th2 cell-related cytokines (IL-4, IL-10) were significantly decreased in PsD mice compared with normal mice in both the WT and CB2R KO groups. Interestingly, CB2R deficiency exhibited the greatest impact on the expression of IL-2 in the serum after applying IMQ, given that the changes in several other cytokines were not statistically significant (Figure 2C). These results indicated that CB2R deficiency exacerbated the IMQ-induced skin inflammation by regulating inflammatory cell infiltration and the release of local and systemic inflammatory cytokines.

### CB2R Deficiency Promoted the Differentiation Proportion of Th17/Treg Cells Among CD4^+^ T Cells

**FIGURE 3:**
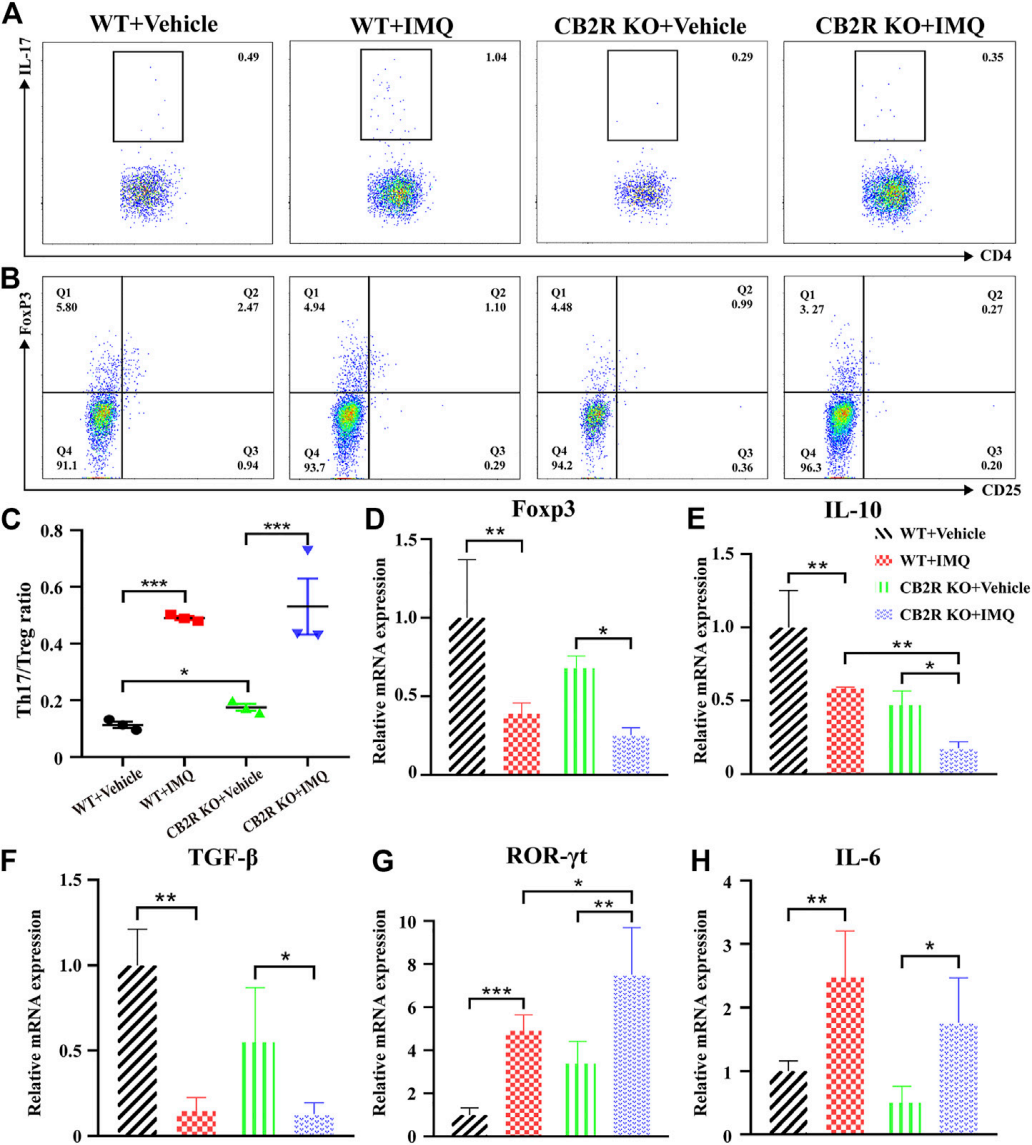
Effects of CB2R deficiency on differentiation proportion of Th17/Treg in IMQ-induced PsD. **(A)** Flow cytometric analysis of the proportion of Th17 cells (CD4^+^, IL-17A^+^ cells) in single cell suspensions of spleen of IMQ- and vehicle- treated skin in WT and CB2R KO mice. **(B)** Flow cytometric analysis of the proportion of Treg cells (CD4^+^, CD25^+^, and Foxp3^+^ cells) in single cell suspensions of spleen of IMQ- and vehicle- treated skin in WT and CB2R KO mice. **(C)** The ratio of Th17/Treg cells. **(D-H)** The mRNA expression levels of Foxp3 **(D)**, IL-10 **(E)**, TGF-β **(F)**, ROR-γt **(G)**, IL-6 **(H)**. Error bars represent mean ± SD. *p < 0.05, **p < 0.01, and ***p < 0.001 when compared.

It is well known that psoriasis may result from massive inflammatory cellular infiltrates and/or an imbalance in T cells, especially in Th17/Treg cells (Shi et al., 2019). Furthermore, it has been reported that CB2R activation may change the balance of Th17/Treg cells in lung tissues (Wei et al., 2021), but it remains unclear whether it plays the same role in psoriasis. To probe the effect of CB2R in regulating the Th17/Treg cell balance on IMQ-induced systemic inflammation, we next performed flow cytometric analysis of splenic single cells. We found that the proportion of Th17/Treg cells was increased in IMQ-induced PsD mice compared with vehicle-induced control mice. However, CB2R deficiency slightly increased the ratio of Th17/Treg cells in the spleen compared to that of WT mice after IMQ application. However, this effect was more obvious in vehicle mice (Figures 3A–C). We also verified the mRNA expression levels of ROR-γt, TGF-β, IL-6, Foxp3 and IL-10, which play a critical role in the differentiation of Th17 and Treg cells, respectively. Importantly, the mRNA levels of Treg-related transcription factors and cytokines, including Foxp3, IL-10, and TGF-β, were significantly decreased in CB2R KO-PsD mice compared with CB2R KO-vehicle mice (Figures 3D–F). In contrast, Th17-related transcription factors and cytokines, including ROR-γt and IL-6, were significantly increased in the two groups (Figures 3G,H). Compared with WT mice, CB2R deficiency had a more obvious effect on IL-10 and ROR-γt expression after applying IMQ. In summary, the experiments demonstrated that CB2R deficiency promoted the proportion of Th17/Treg cells among CD4^+^ T cells.

### The CB2R Agonist JWH-133, was Effective Against IMQ-Induced PsD

**FIGURE 4:**
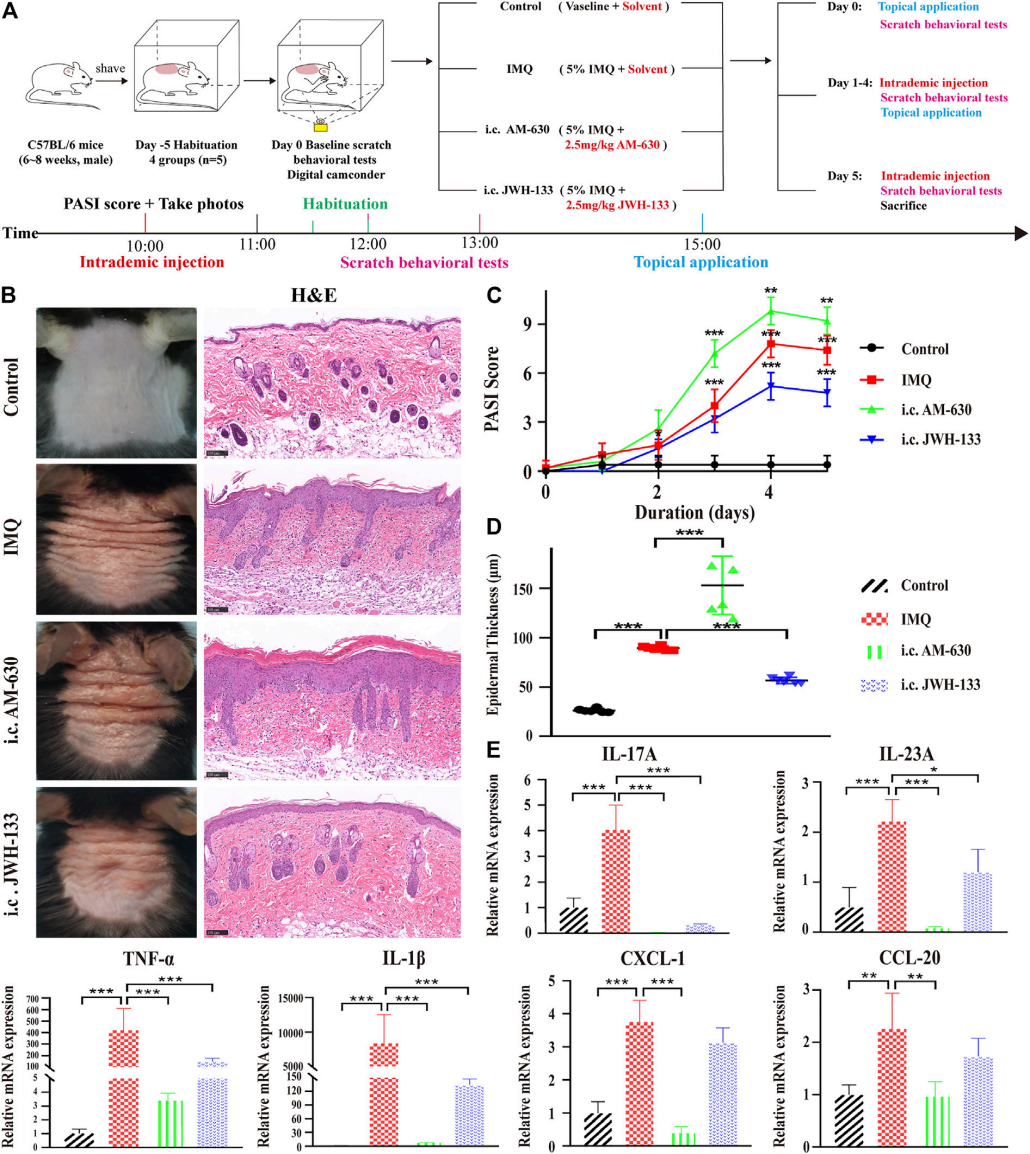
The selective agonist of CB2R, JWH-133, could alleviate PsD in IMQ-induced mice. **(A)** Schematic diagram of the animal experiment protocol for the control, IMQ, i.c. AM-630 and i.c. JWH-133 groups (*n* = 5). **(B)** Representative macroscopic view and H&E staining of cross-sectional slices of the dorsal skin of C57BL/6 mice on the fifth day. Scale bar represents 100 μm. **(C)** Daily assessment of epidermal erythema, scales, and thickening of the dorsal skin. The PASI score was calculated by adding the scores of three independent criteria (ranging from 0 to 12). **(D)** The epidermal thickness of the dorsal skin on the fifth day was measured by four randomly selected fields per section of each mouse. **(E)** The mRNA expression levels of IL-17A, IL-23A, TNF-α, IL-1β, CXCL-1 and CCL-20 were measured by qRT-PCR. Error bars represent mean ± SD. (*n* = 5 for each group). *p < 0.05, **p < 0.01, and ***p < 0.001 when compared. All the assays were repeated three times with consistent results.

We further applied the CB2R agonist JWH-133 and the CB2R antagonist AM-630 to observe their effects on PsD. JWH-133, AM-630 or solvent (10% DMSO+20% Tween-80 + 70% saline) was intraperitoneally (i.p.) injected into WT mice during IMQ treatment. We first found that 5 mg/kg was the optimum dose because 1 and 3 mg/kg were not effective. Intraperitoneal treatment with 5 mg/kg JWH-133 improved the PASI score. Because CB2R was mainly located on the epidermis, then we observed that mice received 2.5 mg/kg JWH-133 intracutaneously (i.c.) showed a significant attenuation of psoriasis inflammation compared with those that received an intraperitoneal injection. Thus, an IMQ-induced PsD mouse model and simultaneous intracutaneous injection pretreatment with AM-630 or JWH-133 were conducted to verify the role of CB2R *in vivo* (Figure 4A). Compared with the IMQ group, JWH-133 visibly alleviated the clinical phenotype of IMQ-induced PsD but did not completely eliminate the macroscopic clinical symptoms, which included redness and thickening of the skin reduction. Histological results showed that JWH-133 significantly improved the histologic changes, which showing less parakeratosis and less inflammatory cell infiltration than the IMQ group. In contrast, AM-630 had the opposite effect (Figure 4B). Similarly, the i.c. JWH-133 group showed lower PASI scores (Figure 4C) and less epidermal thickening (Figure 4D). Consistently, JWH-133 also significantly reduced the mRNA expression levels of proinflammatory cytokines, including IL-17A, IL-23A, TNF-α, IL-1β, CXCL-1 and CCL-20, in skin lesions (Figure 4E). Of note, the expression levels of proinflammatory cytokines also decreased after i.c. administration of AM-630. It is possible that the dose was insufficient or the pharmacological effect of the antagonist was not as thorough as knocking out the receptor. These results demonstrated that CB2R activation alleviated inflammation in IMQ-induced PsD.

### CB2R Deficiency Intensified the Scratching Behaviour of IMQ-Induced Psoriatic Mice

**FIGURE 5:**
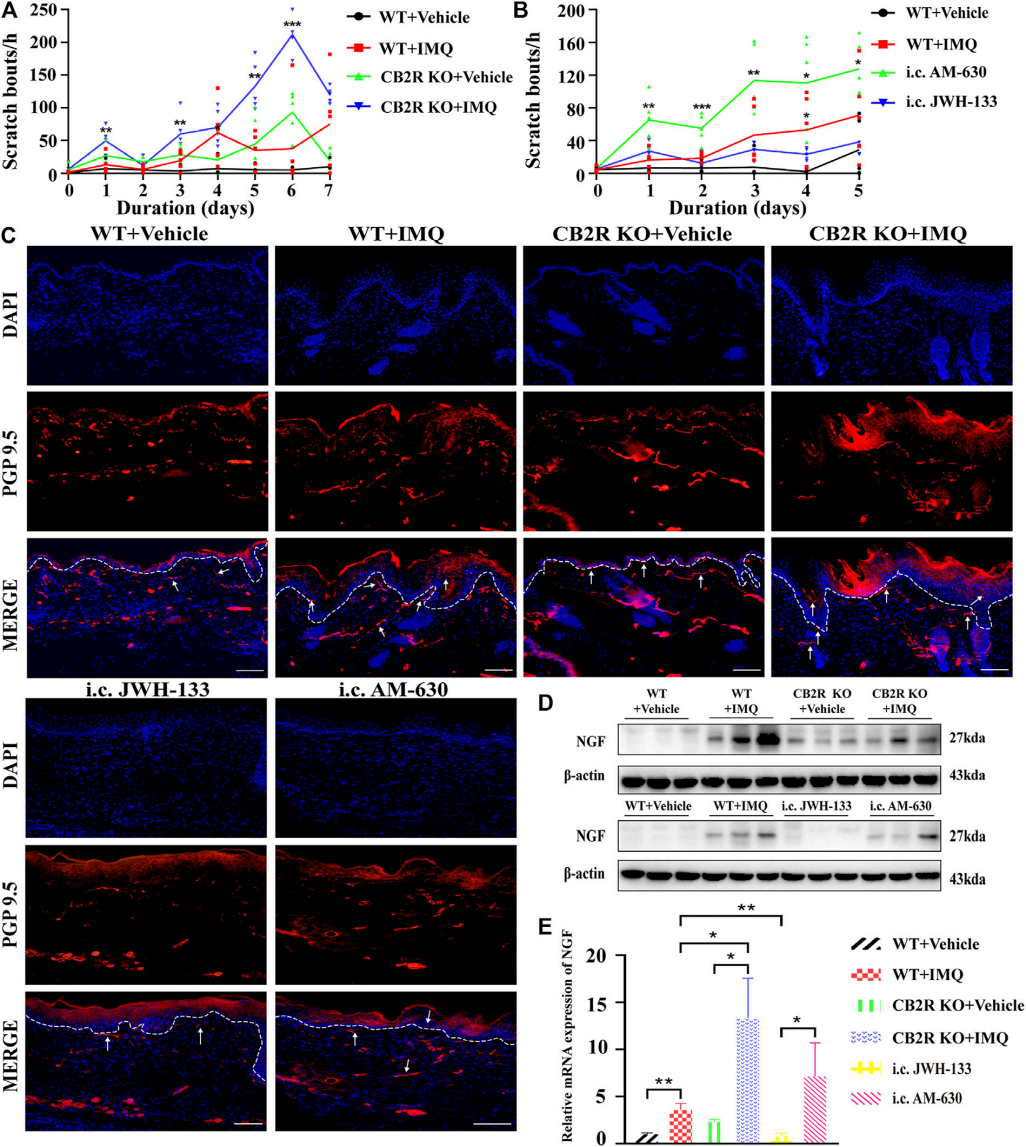
Effects of CB2R on the scratching behavior and expression of nerve fibers in IMQ-induced PsD. **(A, B)** The behavior results of mice in different groups. **(** **C)** PGP 9.5 stained (red fluorescence) nerve fibers in the skin biopsies from mice, co-stained with DAPI (blue fluorescence) to detect the nucleus. Scale bar represents 100 μm. **(D)** Protein level of NGF was analyzed by western blotting in the six groups of mice. β-actin served as the loading control. **(** **E)** The mRNA expression levels of NGF was measured by qRT-PCR. Error bars represent mean ± SD. (*n* = 5 for each group). *p < 0.05, **p < 0.01, and ***p < 0.001 when compared. All the assays were repeated three times with consistent results.

It has been reported that CB2R agonists significantly suppress scratching behaviour induced by compound 48/80, histamine, substance P or serotonin in mice or rats (Odan et al., 2012; Haruna et al., 2015), but all of those compounds are the pruritogens of acute itch. Spontaneous scratching and psoriasiform skin lesions can be induced by topical stimulation of IMQ (Sakai et al., 2016), which simulates the chronic itch of psoriasis. To investigate the effect of CB2R on the chronic itch in psoriasis, we followed the schematic experimental protocol (Figure 4A). The results showed that mice treated with IMQ indeed exhibited more scratching bouts than the control group. Both CB2R deficiency and i.c. AM-630 injection intensified the scratching behaviour, whereas i.c. JWH-133 alleviated the scratching behaviour compared with that in the IMQ model group (Figures 5A,B). We next examined the sensory nerve fibers in lesion skin and untreated skin from WT mice and CB2R KO mice. Nerve fibers density was analyzed using immunofluorescence to visualize protein gene product 9.5 (PGP 9.5)-positive nerve fibers, which are known as peripheral sensory nerve markers (Feld et al., 2016). As shown in Figure 5C and Supplementary Figure S2B, CB2R KO showed that dermal nerve fibers extended to the epidermis, and a significant increase in the density of PGP 9.5-positive nerve fibers at the junction of the dermis and epidermis was noted in IMQ-induced PsD skin compared with WT mice or vehicle-treated skin. To further explore the mechanism of itch, we hypothesized that the changes in nerve fibers involved NGF-induced neuronal growth. Western blot and qRT-PCR revealed an increase in NGF expression levels in the IMQ group compared with the normal control group. However, CB2R KO only affected NGF gene expression level but did not significantly influence NGF protein expression (Figures 5D,E). Taken together, these results suggested that the extension of nerve fibers into the epidermis could be involved in the itch-related scratching of mice, and CB2R deficiency intensified the scratching behaviour of IMQ-induced psoriatic mice by regulating the expression of nerve fibers. Moreover, all of these effects could be reversed by JWH-133, but the effects of the antagonists AM-630 were not as significant as those of the knockout mice.

### The Regulation of CB2R on Psoriatic Itch was Independent of IL-31, TSLP and Mast Cells

**FIGURE 6:**
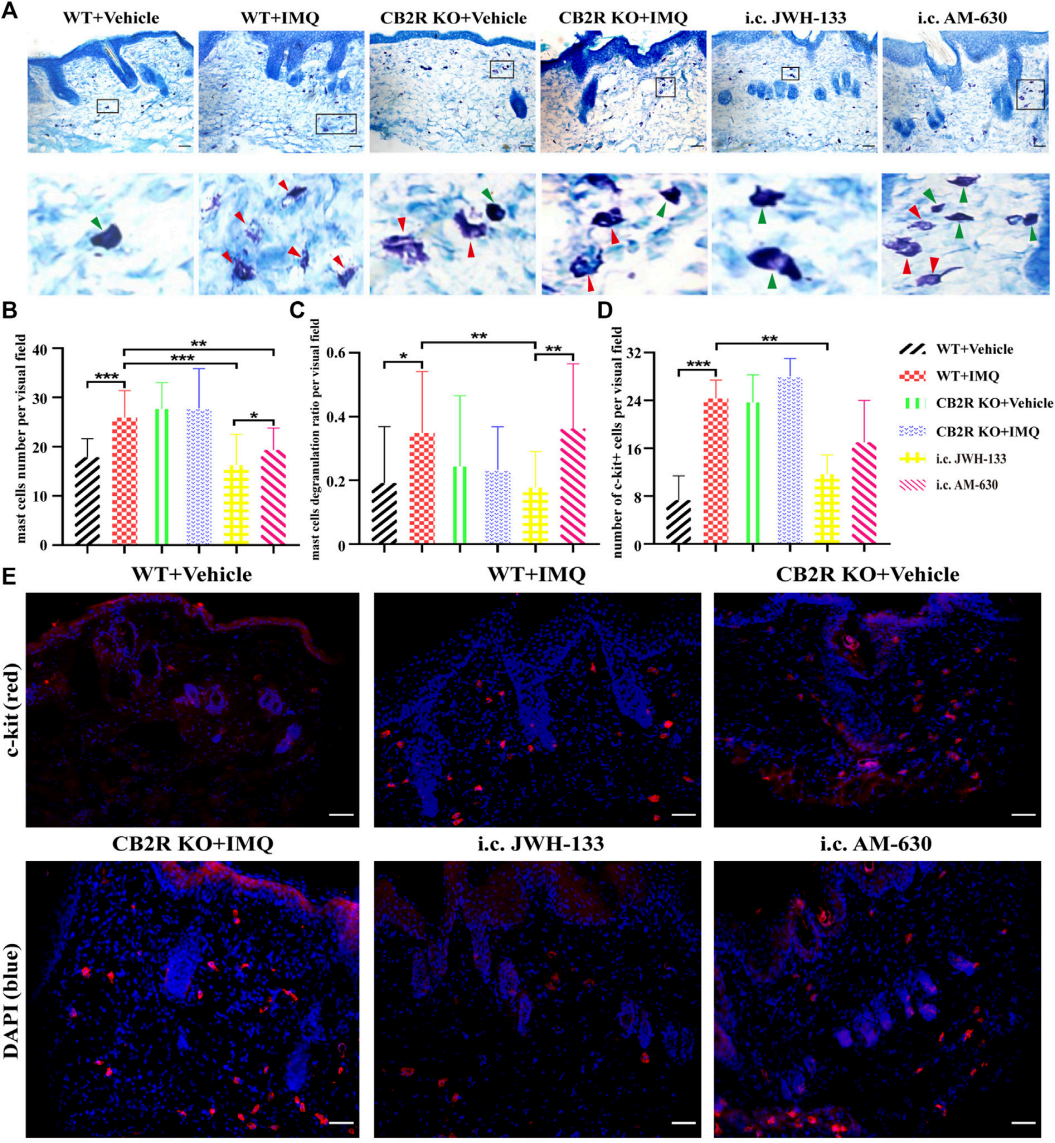
Regulation of mast cells number and degranulation by CB2R in IMQ-induced psoriasis. **(A–C)** Toluidine bule staining to detect the number and the degranulation of mast cells, green arrows pointed to non-degranulated mast cells, red arrows pointed to degranulated mast cells. **(D, E)** Immunofluorescence to analysis of c-kit (mast cell marker) expression and quantification of mast cells number in the skin. Error bars represent mean ± SD. *p < 0.05, **p < 0.01, and ***p < 0.001 when compared. Scale bar represents 100 μm.

IL-31, thymic stromal lymphopoietin (TSLP) and mast cells have recently attracted much attention as targets for chronic itch therapy (Agarwala et al., 2016). However, whether CB2R is involved in psoriatic itch by regulating these cytokines and mast cells remains unclear. We used immunofluorescence to label the mast cells surface marker c-kit and toluidine lube staining to observe the changes in the number and activation of mast cells in the skin. As shown in Figures 6A–E, the infiltration and degranulation rate of mast cells increased after IMQ treatment in WT mice. However, we found no difference in mast cells between IMQ-treated CB2R KO mice and WT mice. Interestingly, CB2R KO significantly reduced the mRNA expression of IL-31 and TSLP after IMQ application compared with WT mice (Supplementary Figures S2C,D), showing an opposite trend to scratching bouts. Intradermal JWH-133 reversed the expression of IL-31and TSLP and the activation of mast cells in PsD mice. Collectively, these data suggested that CB2R didn’t participate in the induction of itch in psoriasis by regulating the expression of IL-31, TSLP and mast cells in mice.

## Discussion

In recent years, an emerging concept has been proposed that the skin serves as a neuro-endocrine-immune organ to maintain global and local homeostasis through multidirectional communication among the brain, the endocrine and immune systems, and peripheral organs (Slominski and Wortsman, 2000). This homeostasis can be unbalanced due to environmental stress, trauma and metabolic disorders, underscoring the pathogenesis of some skin diseases, such as atopic dermatitis and psoriasis (Slominski et al., 2012; Slominski et al., 2013). It is known that the pathogenesis of psoriasis is a result of complex interactions among the neuroendocrine, immune and vascular systems (Armstrong and Read, 2020). Evidence suggests that CB2R plays a critical role in immune modulation and sensory conduction (Sheriff et al., 2020; Graczyk et al., 2021). Although CB2R-mediated immune responses have been observed in other inflammatory skin diseases, including skin wound healing (Wang et al., 2016; Du et al., 2018), allergic contact dermatitis (Karsak et al., 2007) and artificially induced dermatitis (Haruna et al., 2017), little is known about their role in psoriasis. We first found that CB2R expression levels were significantly increased in psoriatic lesions compared with normal skin. CB2R was mainly expressed in the suprabasal epidermis, indicating that endocannabinoid signaling is implicated in the regulation of the epidermal permeability barrier homeostasis (Roelandt et al., 2012). Positive staining for CB2R was also observed in the dermis, which may be due to it located in cutaneous nerve fiber bundles, mast cells, macrophages, the epithelial cells of hair follicles, sebocytes and eccrine sweat glands (Ständer et al., 2005). These findings were consistent with the concept that CB2R expression increased by greater than 100 times the base level due to the inflammation (Lu and Mackie, 2016). CB2R overexpression in psoriatic skin tissues may be a synergistic action of acanthosis cell layer thickening, or it may be a self-protective response of reactive upregulation to inhibit keratinocyte proliferation. CB2R deficiency unexpectedly exacerbated psoriasiform skin inflammation.

The balance of Th17/Treg cells plays a critical role in the pathogenesis of psoriasis. Some studies have shown that Treg cells are easily converted into Th17 cells under psoriatic inflammatory conditions (Bovenschen et al., 2011). Shi et al. confirmed the existence of an imbalance of Th17/Treg cells in psoriasis patients (Shi et al., 2019). CB2R is expressed in B lymphocytes, neutrophils, leucocytes CD8 and CD4, allowing cannabinoids to mitigate the inflammatory response (Graczyk et al., 2021). A previous *in vitro* study found that the CB2R selective agonist O-1966 activated Tregs and promoted increased IL-10 secretion in the mixed lymphocyte reaction culture supernatant (Robinson et al., 2015). In the present study, we found that CB2R KO contributed to the pathogenesis of psoriasis by promoting CD4^+^ T cell proliferation; upregulating Th17 cytokines, such as IL-17A, IL-23A, and IL-17C; and downregulating IL-10. In addition, CB2R KO aggravated the imbalance of Th17 and Treg cells by regulating the expression of key transcription factors ROR-γt and Foxp3. The changes in these cytokines at the protein level were confirmed by CBA assay. The protein results showed the same trends as the mRNA results, suggesting that CB2R has a regulatory effect on systemic and local inflammation. Thus, future studies should focus on the molecular mechanism of CB2R activation in regulating CD4^+^ T cell differentiation at the cellular level as a more representative comparison to our murine studies.

Although it has been reported that a variety of endogenous and exogenous CB2R agonists can suppress acute itch-associated scratching behaviour in rodents through inhibition of itch signal transmission (Haruna et al., 2015; Avila et al., 2020), the relationship between CB2R and chronic itch is poorly understood, especially in psoriasis itch. Previous studies have demonstrated that the increase in nerve fiber density and the prolongation of nerve fiber endings to the epidermis could promote itch hypersensitivity, which subsequently leads to psoriasis pruritus (Tominaga et al., 2007). We found that the immunoreactive nerve fibers treated with anti-PGP 9.5 antibody were markedly increased in the epidermis of IMQ-induced WT and CB2R deficiency mice on Day 7. Thus, these results appear to indicate that CB2R KO could lead to increased nerve ending density and length, and the changes in nerve fibers may be related to the frequency of scratching in psoriatic mice. Our work also provided evidence for the involvement of CB2R in the regulation of chronic itch.

The mechanisms by which nerve fibers extend in psoriatic mice and CB2R deficiency modulates itching remain unclear. Various studies provide evidence that some neurotrophins can contribute to neural extension (Menezes et al., 2019; Sultan et al., 2021). NGF is abundantly released from human keratinocytes in culture, and is highly expressed in the epidermis of psoriatic mice (Pincelli et al., 1994). Some researchers have observed that compared with nonpruritic skin, NGF content in pruritic psoriasis skin lesions was increased (Yamaguchi et al., 2009) and accompanied by increased nerve density in the skin (Tan et al., 2019). Furthermore, NGF expression in astrocytes and microglia was markedly upregulated by local tissue injury, inflammation and cytokines (Pöyhönen et al., 2019). Bozkurt et al. reported that CB1R activation could continuously modify neuronal airway hyperreactivity by inhibiting neuronal growth induced by NGF (Bozkurt et al., 2016). Whether CB2R has a similar effect on NGF expression has not yet been reported. Our results showed that NGF expression levels were significantly upregulated in psoriasis skin lesions. CB2R deficiency significantly increased NGF mRNA but not protein expression, and the translation of mRNA to protein might be inhibited by CB2R. We assumed that in pruritus psoriasis skin, the number of nerve fibers increases, and the new nerve fibers promote the continued secretion of NGF, resulting in abnormal hyperplasia of nerve fibers and more sensitive sensation. In addition, the local high expression of a variety of inflammatory factors for NGF secretion and nerve fiber hyperplasia are also effective stimuli, leading to pruritus paresthesia. Together, NGF expression and nerve fiber changes may represent the essential changes that are responsible for itching in CB2R-deficient psoriatic mice. However, there may be other factors in addition to NGF that cause nerve fiber changes and itch in psoriasis. The mechanism by which CB2R regulates NGF expression also needs to be further elucidated.

CB2R is mainly present in immune cells such as mast cells (Rom and Persidsky, 2013), and the latter have been implicated as key mediators of itch (Siiskonen and Harvima, 2019). Although it has been reported that the number and degranulation of mast cells elevated in pruritic psoriatic lesions (Harvima et al., 2008; Cho et al., 2017), lack of study investigate the correlation between CB2R and mast cells in psoriasis. Our data demonstrated that there was indeed an increase in the number and degranulation of mast cells in PsD. CB2R deficiency increased mast cells activation in normal control mice, but had no effect on IMQ-induced psoriasis mice. IL-31 and TSLP are key cytokines directly promoting itch (Dillon et al., 2004; Wilson et al., 2013). Recent studies demonstrated that IL-31 and TSLP appear to be partially involved not only in the pathogenesis of psoriasis, but also in the induction of itch in psoriasis (Nattkemper et al., 2018; Gago-Lopez et al., 2019; Gibbs et al., 2019; Suwarsa et al., 2019). Our study was consistent with previous findings in WT mice. However, CB2R deficiency contributed to opposite results. Combined with the results of i.c. JWH-133 group, we cannot exclude the possibility that location of CB2R in other cells other than keratinocytes and mast cells might be involved in the pathogenesis. The generation of tissue - or cell-specific knockout CB2R mice is necessary for further studies.

**FIGURE 7:**
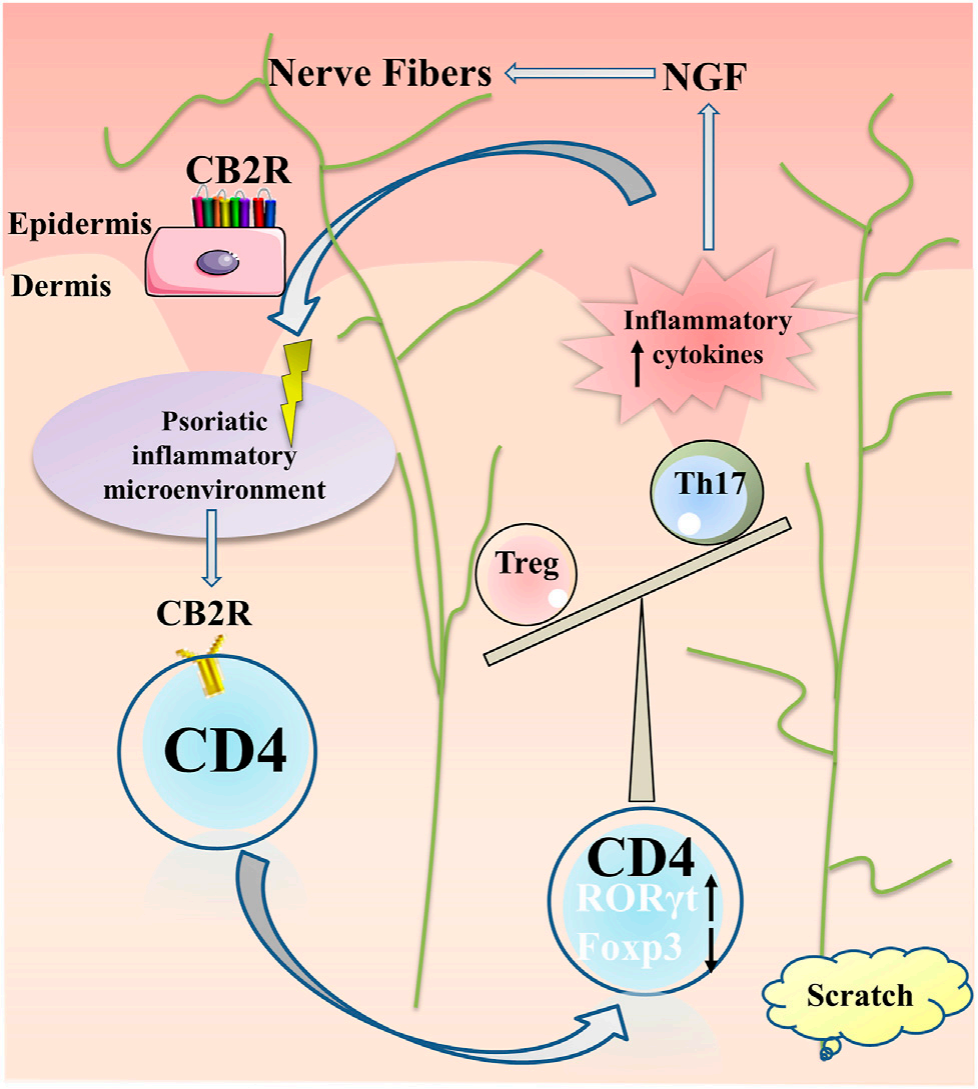
Summary of CB2R actions in psoriatic inflammation and itch. Under the psoriatic inflammatory microenvironment, CB2R regulated proliferation and differentiation of CD4^+^ T cell, increased ROR-γt expression and decreased Foxp3 expression, resulting in imbalance of Th17 and Treg cells, further aggravating the inflammatory microenvironment of psoriasis. The local high expression of a variety of inflammatory factors stimulated the keratinocytes secreting NGF for nerve fiber hyperplasia, resulting in pruritus paresthesia.

In conclusion, our results suggested that CB2R may play a protective role in mediating immunosuppression by regulating the proliferation and differentiation of CD4^+^ T cells and reducing the production of cytokines and chemokines. In addition, the reduction of the release of inflammatory factors may weaken the NGF secreted by keratinocytes, reduce the proliferation and elongation of nerve fibers, and have an antipruritic effect (Figure 7). The neuro-immune pathway may be involved in the regulation of inflammation and itch in psoriasis. Given CB2R’s localized expression, most of the adverse side effects associated with systemic therapy can be avoided. Topical preparations are readily absorbed through the skin, which is traditionally the preferred route of delivery for treating psoriasis. These considerations have led to the hypothesis that topical selective CB2R agonists may have potential therapeutic applications for the treatment of psoriasis.

## Data Availability

The original contributions presented in the study are included in the article/Supplementary Material, and at the following link, further inquiries can be directed to the corresponding authors: https://www.jianguoyun.com/p/Db79a-YQuImUChjCoqYE.
