# Supplementary material for: CB2R Deficiency Exacerbates Imiquimod-Induced Psoriasiform Dermatitis and Itch Through the Neuro-Immune Pathway
Source: Front Pharmacol. 2022 Jan 31;13:790712. doi: 10.3389/fphar.2022.790712 (PMC8841964; doi:10.3389/fphar.2022.790712)
Supplement: Supplementary file 1 [file Table1.docx]

**Table S1. Antibodies information**

| **Antibodies** | **Source** | **Concentration** | **Application** |
| --- | --- | --- | --- |
| Anti-Rabbit CD4 | Abcam | 1: 1000 | IHC |
| Anti-Rabbit CB2R | Abcam | 1: 200 | IF |
| Anti-Goat c-kit | RD System | 1: 500 | IF |
| Anti-Rabbit PGP 9.5 | ABclonal | 1: 500 | IF |
| Anti-Rabbit CB2R | ABclonal | 1: 500 | WB |
| Anti-Rabbit NGF | Affinity | 1: 500 | WB |
| Anti-Rabbit β-actin | ABclonal | 1: 20000 | WB |
| BV510 Rat Anti-Mouse CD4 | BD Pharmingen | 1: 400 | FCM |
| PerCP-Cy5-5 Rat Anti-Mouse CD3e | BD Pharmingen | 1: 1000 | FCM |
| PE Rat Anti-Mouse CD25 | BD Pharmingen | 1: 400 | FCM |
| BV421 Rat Anti-Mouse IL-17A | BD Pharmingen | 1: 400 | FCM |
| Alexa Fluor 647 Rat Anti-Mouse Foxp3 | BD Pharmingen | 1: 400 | FCM |
