# Supplementary figures and images for: CB2R Deficiency Exacerbates Imiquimod-Induced Psoriasiform Dermatitis and Itch Through the Neuro-Immune Pathway
Source: Front Pharmacol. 2022 Jan 31;13:790712. doi: 10.3389/fphar.2022.790712 (PMC8841964; doi:10.3389/fphar.2022.790712)

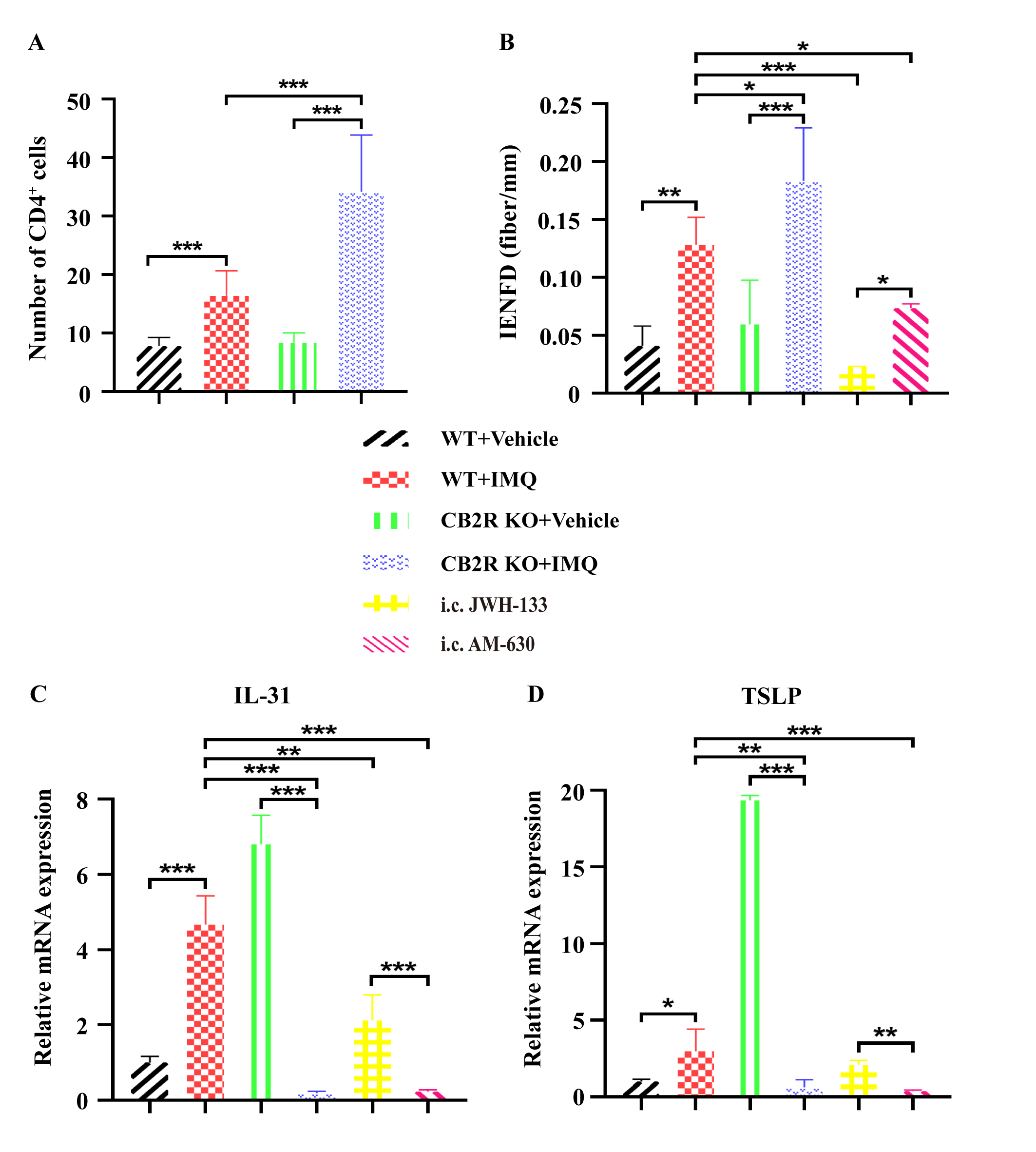

Supplement: Supplementary file 2 [file Image2.TIF]

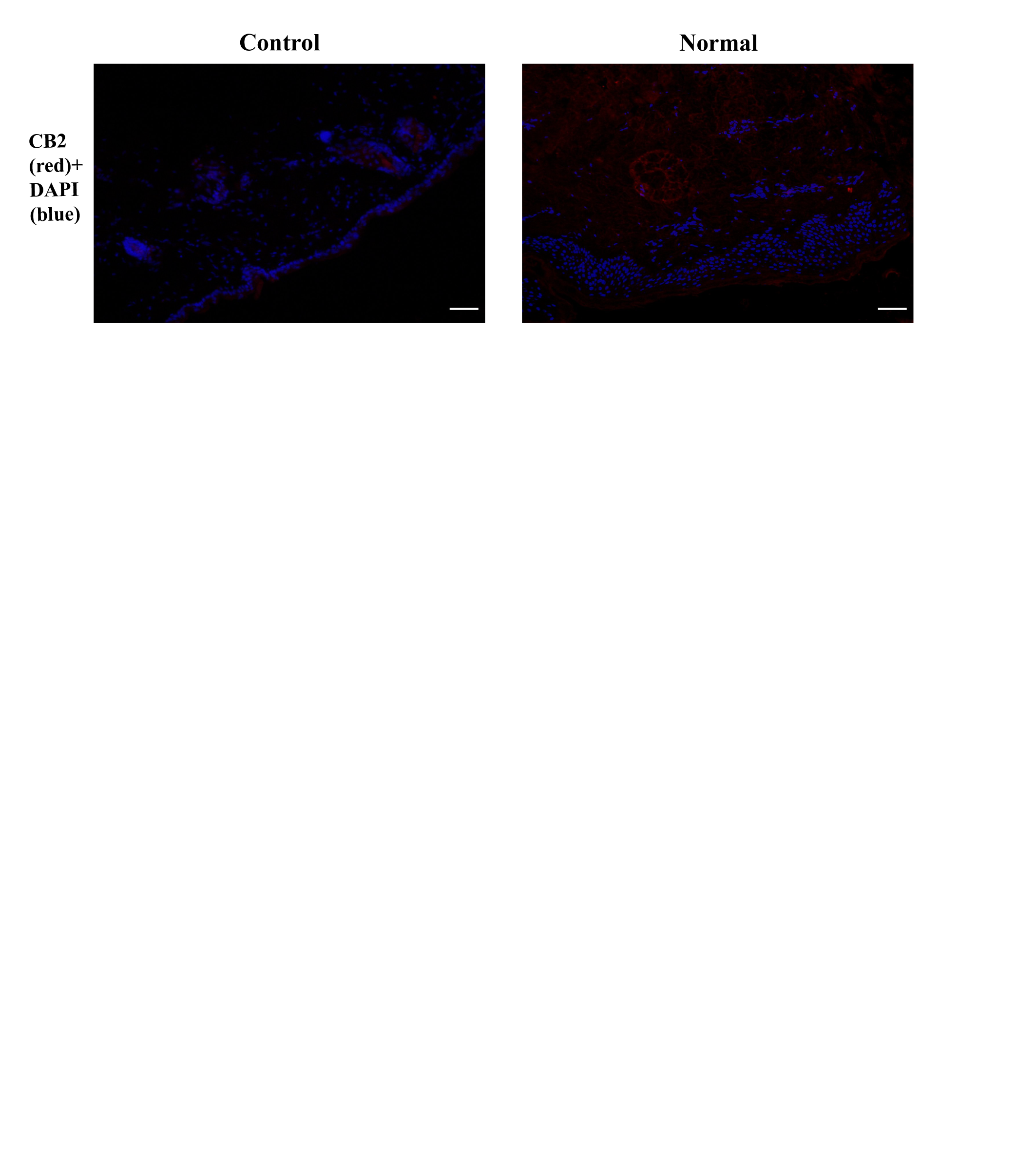

Supplement: Supplementary file 3 [file Image1.TIF]
